# Current Topics in Esophageal Squamous Cell Carcinoma

**DOI:** 10.3390/cancers12102898

**Published:** 2020-10-09

**Authors:** Hiroki Kurumi, Hajime Isomoto

**Affiliations:** Division of Gastroenterology and Nephrology, Department of Multidisciplinary Internal Medicine, Tottori University Faculty of Medicine, 36-1 Nishicho, Yonago 683-8504, Japan; kurumi_1022_1107@yahoo.co.jp

**Keywords:** esophageal squamous cell carcinoma, intensity-modulated radiation therapy, topoisomerase 1, stromal cell-derived factor-1α, PET-CT, sarcopenia

## Abstract

Esophageal squamous cell carcinoma (ESCC) is one of the most deadly cancers due to its extremely aggressive nature and poor survival rate. Central to East Asia is one of regions with the highest incidence of ESCC. In these five papers, the international leaders of ESCC in Asia have taken various approaches to ESCC. Lin et al. compared intensity-modulated radiation therapy with three-dimensional stereoscopic radiation therapy with respect to treatment of ESCC. Song et al. demonstrated that (S)-10-Hydroxycamptothecin is useful in the ESCC cell lines as well as in vivo using a patients-derived xenograft tumor model in mice. Chen et al. showed Stromal cell-derived factor-1α expression is an independent prognostic predictor of ESCC. Lin et al. showed that the SUV_LN_/SUV_Tumor_ ratio of PET-CT was associated with ESCC prognosis. Yoon et al. investigated the association between sarcopenia and prognosis in the ESCC patients. All reports are an essential approach to overcoming ESCC.

Approximately 604,000 new cases of esophageal cancers (EC) are expected to be diagnosed in 2020, resulting in 537,000 cancer deaths worldwide. EC is the seventh most common malignancy in terms of incidence and the sixth leading cause of cancer death worldwide [1]. The histological type of EC varies widely by geographic distribution, with more than 10-fold disparities between regions. Central to East Asia is one of the regions with the highest incidence of esophageal squamous cell carcinoma (ESCC) [2]. ESCC is one of the most deadly cancers in the world due to its extremely aggressive nature and poor survival rate. For ESCC not indicated for endoscopic resection, a combination therapy of surgery, chemotherapy, and radiotherapy is chosen, depending on the American Joint Committee on Cancer (AJCC)/International Union Against Cancer (UICC) clinical stage and patient background. Despite the recent development of multimodality therapies such as surgery, chemotherapy, and radiotherapy, the prognosis of ESCC patients remains poor [3]. Therefore, the efforts to improve the prognosis of ESCC are an urgent issue. In these five papers, the international leaders of ESCC in Asia have taken various approaches to ESCC. 

Lin et al. focused on radiotherapy. Radiotherapy plays an important role in the treatment of both respectable and unresectable EC. They compared intensity-modulated radiation therapy (IMRT) with three-dimensional stereoscopic radiation therapy (3D-RT) with respect to the treatment of ESCC. IMRT is expected to be more effective than 3D-RT in terms of target coverage, dose uniformity, and reduced toxicity to normal organs [4]. Several reports suggest that an IMRT-delivered high radiation dose produces significantly lower average percent volumes of irradiated adjacent organs than 3D-RT [5,6]. They found that in the 2062 patients who received chemoradiotherapy (CRT) against the thoracic esophageal squamous cell carcinoma, IMRT significantly improved overall survival (OS) compared to 3D-RT (aHR: 0.88, 95% CI: 0.78–0.98, *p* = 0.022) and was more pronounced in advanced stages (ⅢA–IIIC according to the AJCC )(aHR: 0.88, 95% CI: 0.77–0. 99, *p* = 0.036).

The reports by Song et al. showed the potential for new drugs against ESCC in their report. Although taxane-, cisplatin-, and fluorouracil-based chemotherapy is common for the treatment of patients with advanced or metastatic ESCC, there are not enough treatment options, and furthermore, the prognosis remains poor [3,7]. Recently, several clinical studies have reported promising efficacies and manageable safety profiles of anti-PD-1 antibodies in patients with advanced or metastatic ESCC [8,9]. In several countries, anti-PD-1 antibodies are approved for the treatment of EC. In this study, they focused on the Topoisomerase 1 (TOP 1). A TOP 1 is often exploited as an imperative anticancer chemotherapeutic target due to its critical role in DNA supercoil relaxation [10,11]. The camptothecin (CPT) family has anticancer effects by selectively inhibiting TOP1. (S)-10-Hydroxycamptothecin (HCPT), a nature CPT analog that is more potent and less toxic than irinotecan, has recently undergone extensive evaluation worldwide [12]. They demonstrated that HCPT is useful in the ESCC cell lines as well as in vivo using a patients-derived xenograft tumor model in mice. Although there are some problems to be solved, such as poor water solubility and internal instability caused by the opening of the labile lactone ring at physiological pH, it could be a potential new agent for ESCC [12].

Chen et al. focused on a chemokine called Stromal cell-derived factor-1α (SDF-1α) and its receptor, C-X-C chemokine receptor type 4 (CXCR4). These signaling pathways have been reported to play a pivotal role in the growth and metastasis of cancers such as gastric, colorectal, and pancreatic cancer [13,14], but their relevance to EC has been controversial. They demonstrated that AMD3100 (a CXCR 4 antagonist) suppressed tumor growth in a dose-dependent manner in vitro. Furthermore, they examined the association between the expression of SDF-1α and the prognostic value, disease-free survival (DFS), and OS in 169 surgically treated patients with ESCC (Stage I/II/III/IVA: 51/63/34/21 according to the AJCC). Multivariate analysis showed SDF-1α expression is an independent prognosis predictor of DFS and OS. SDF-1α may be used as a biomarker for high-risk groups with a poor prognosis in their study.

Lin et al. also found out about the potential usefulness of screening high-risk groups. They examined the SUV_LN_/SUV_Tumor_ ratio of PET-CT taken before treatment in 112 patients with clinically node-positive ESCC (Stage IIB/IIIA/IIIB/IIIC:3/13/25/71 according to AJCC) who had undergone definitive CRT. In their study, the patients with a high SUV_LN_/SUV_Tumor_ ratio (>0.39) correlated with a poor distant metastasis-free survival (DMFS) and OS (two-year DMFS: 26% vs. 70%, *p* < 0.001; two-yars OS: 21% vs. 48, *p* = 0.001). Identification of the poor prognosis group distinguished the patients who needed frequent follow-up from those who needed more intensive treatment.

Yoon et al. focused on the patients’ backgrounds. They investigated the association between sarcopenia and prognosis in the ESCC patients who underwent neoadjuvant CRT followed by surgery. Sarcopenia is now formally recognized as a muscle disease with an ICD-10-MC Diagnosis Code that can be used to bill for care in some countries. In its 2018 definition, European Working Group on Sarcopenia in Older People used low muscle strength as the primary parameter of sarcopenia; muscle strength is presently the most reliable measure of muscle function [15]. They retrospectively examined 248 patients and found that sarcopenia defined as skeletal muscle index (SMI) < 52.4 cm^2^/m^2^ was not associated with OS. However, the excessive muscle loss defined as SMI change <−10.0% was related to the OS. It is suggested that proper management of nutritional status during treatment appears to be more important than pre-treatment malnutrition. Patients with advanced EC tend to suffer from malnutrition with reduced oral intake due to cancer-related stricture and other factors. Because of the invasive nature of cancer treatment, it is important to take steps to prevent deteriorating nutritional status during treatment.

In addition to the above, various other efforts are being made around the world to overcome EC. Systematic treatment is important to defeat EC, and there is a need to develop new approaches as well as improve existing ones.
